# The effect of anxiety on sleep disorders in medical students: a moderated mediation model

**DOI:** 10.3389/fpsyg.2024.1338796

**Published:** 2024-03-11

**Authors:** Chuang Yu, Zhiyi Liu, Tiehong Su, Zhongyu Li, Zinan Jiang, Wen Zhong, Zhongju Xiao

**Affiliations:** ^1^Department of Psychology, School of Public Health, Southern Medical University, Guangzhou, China; ^2^Department of Physiology, School of Basic Medical Sciences, Key Laboratory of Psychiatric Disorders of Guangdong Province, Guangdong-Hong Kong-Macao Greater Bay Area Center for Brain Science and Brain-Inspired Intelligence, Key Laboratory of Mental Health of the Ministry of Education, Southern Medical University, Guangzhou, China; ^3^School of Biomedical Engineering, Southern Medical University, Guangzhou, China; ^4^School of Traditional Chinese Medicine, Southern Medical University, Guangzhou, China; ^5^General Practice Center, The Seventh Affiliated Hospital, Southern Medical University, Foshan, China

**Keywords:** anxiety, sleep disorders, flourishing, neuroticism, moderated mediation

## Abstract

The relationship between anxiety and sleep disorders is a key research topic in the academic community. However, evidence on the mechanism through which anxiety influences sleep disorders remains limited. The purpose of this study was to investigate the roles of flourishing and neuroticism in the mechanism through which anxiety influences sleep disorders in medical students. We constructed a moderated mediation model and tested the mediating role of flourishing and the moderating role of neuroticism in medical college students. The results showed that: (1) anxiety was significantly and positively related to sleep disorders and significantly and negatively related to flourishing; flourishing was significantly and negatively related to sleep disorders; neuroticism was significantly and positively related to sleep disorders; (2) flourishing had a mediation effect on the relationship between anxiety and sleep disorders; (3) neuroticism moderated the process through which flourishing mediated the effect of anxiety on sleep disorders. Our research expands the literature on the mechanism underlying the effects of anxiety on sleep disorders and provides insights into the potential prevention and intervention of sleep and emotional problems in medical students.

## 1 Introduction

China National Mental Health Survey, the prevalence rate of anxiety disorders in China is 7.6% (Huang et al., 2019). Anxiety is a complex emotional and psychological response that originates from feelings such as tension, fear, sadness, worry, and panic. Freud believed that anxiety is caused by a lack of security during childhood and adulthood. Research has shown that anxiety-related psychopathology seems to be associated with feelings of uncertainty and insecurity (Suarez et al., 2008). Anxiety is not produced in response to current, identifiable threats but rather in response to unresolved or potential threats that may or may not occur (Zhou et al., 2022). Therefore, anxiety seems to imply uncertainty, lack of control, and insecurity about the future. With the continuous acceleration of urbanization, medical students’ interpersonal relationships have become increasingly complicated, and competition has become more intense. Faced with uncertainty over the future, medical students are prone to stress, anxiety, and other negative emotional experiences (Tanya et al., 2022).

One of the negative impacts of anxiety can be the development of sleep disorders. Anxiety can predict sleep disorders, and sleep disorders are also important risk factors for anxiety and depression (Choueiry et al., 2016; Hertenstein et al., 2019). Some studies emphasize the bidirectional relationship between emotions and sleep, leading to a vicious cycle of sleep difficulties. Studies have shown that medical students in China have lower sleep quality compared to non-medical students and the general population, which may have adverse effects on their academic performance, physical and mental health, and quality of life (Saravanan and Wilks, 2014; Zheng et al., 2016). Sleep disorders can lead to decreased immunity, reduced adaptability to life, anxiety, depression, and other issues (Zhang, 2015). The sleep characteristics of college students include irregular sleep patterns, insufficient sleep, and poor sleep quality (Lund et al., 2010; Galambos et al., 2013), and the sleep quality of medical students is even more concerning (Feng et al., 2005; Zheng et al., 2016). To date, research on sleep disorders and anxiety has mostly focused on negative emotions, with relatively little exploration of the relationship between sleep disorders and positive psychological energy.

In exploring the mechanism through which anxiety influences sleep disorders, previous studies have demonstrated the mediating roles of bedtime procrastination, fear and rumination in the relationship between anxiety and sleep disorders (Pace-Schott et al., 2015; Campbell and Bridges, 2023; Faccini et al., 2023). However, the role of positive psychological energy, such as flourishing, has often been ignored. As an exception, Gyasi et al. (2023) focused on happiness in exploring the relationship between anxiety and sleep disorders. In their study, they examined the negative association between sleep problems and happiness.

In the current study, we aimed to find a positive psychological factor that could alleviate anxiety and sleep disorders. We focused on flourishing, which reflects the ability of self-control and the regulation of thoughts and emotions (Mirandi et al., 2023). Whether an individual can correctly control and regulate their emotions to some extent affects their subsequent emotional experience and sleep quality (Guarana et al., 2021). Our study aimed to investigate the psychological processing mechanism by which anxiety influences sleep disorders from the perspective of flourishing.

The impact of anxiety on individuals is modulated by personal traits, such as neuroticism. Compared to individuals with stable personalities, individuals who exhibit neuroticism may be more prone to self-control problems due to anxiety. Examining the psychological traits that may serve as moderators in the process by which anxiety affects sleep disorder through flourishing can deepen our understanding of the conditions under which anxiety has stronger or weaker effects.

In summary, we sought to analyze the pathways and conditions through which anxiety influences sleep disorders from the perspective of positive psychology and provide practical insights for improving the sleep quality of medical students. Current research has mainly focused on the relationship between anxiety and sleep (Guo et al., 2016), and there is an urgent need to explore the mediating effects (how anxiety affects sleep disorders) and moderating effects (under what conditions anxiety has a stronger or weaker impact on sleep disorders).

## 2 Literature review and hypotheses

### 2.1 Anxiety and sleep disorders

Sleep is a necessary physiological and psychological process for individuals to maintain their daily activities. It is an important state in which the body can recover, integrate, and consolidate memories (Krueger et al., 2016). Good sleep quality is essential for normal physical and mental functioning, while prolonged sleep problems can impair cognitive function and increase feelings of stress, emotional issues, physiological disorders, and suicide risk (Guo et al., 2016; Ahmad and Bashir, 2017; Amaral et al., 2018; Bouchoucha et al., 2018; Wu et al., 2019). Research has shown that sleep quality is closely related to the academic performance, physical and mental health, and emotions of college students (Chen and Chen, 2019). Epidemiological studies show that sleep disturbances, particularly insomnia, affect approximately 50% of individuals with anxiety, and that insufficient sleep can instigate or further exacerbate it (Chellappa and Aeschbach, 2022). Growing molecular imaging evidence posits that specific neurotransmitter mechanisms underlying sleep–wake regulation, such as the adenosinergic system, are involved in anxiety (Hohoff et al., 2014; Elmenhorst et al., 2017; Hohoff et al., 2020). Therefore, we proposed the following hypothesis:

*Hypothesis 1*: Anxiety is positively related to sleep disorders.

### 2.2 The mediating role of flourishing

Flourishing does not entail being free from illness and troubles, and nor is it about pure happiness and joy. A high level of flourishing means living in an ideal human society, incorporating kindness, reproduction, growth, and resilience (Sharma, 2016). Flourishing surpasses other indicators of wellbeing, encompassing important aspects of human functioning such as positive relationships, feelings of competence, and having meaning and purpose in life (Diener et al., 2010).

The emotion security hypothesis suggests that individuals experiencing anxiety may experience uncertainty and insecurity about the future, which in turn influences their sense of wellbeing (Davies and Cummings, 1994) and level of flourishing (Kelly-Hedrick et al., 2023). Individuals with anxiety tend to have low levels of flourishing, which affects their symptomatic and functional recovery (Kelly-Hedrick et al., 2023). Furthermore, the Broaden-and-Build Theory (Fredrickson, 2004) of positive emotions suggests that positive psychological resources such as pleasure, flourishing, and happiness can broaden an individual’s momentary thought–action repertoire, facilitating the development of physical and psychological resources. Conversely, negative emotions hinder the development of physical and mental resources (Yin, 2021). Physical resources include skills and health, with sleep quality being an important aspect of physical health (Perotta et al., 2021). Armand et al. argued that an individual’s level of flourishing can impact both the duration and quality of sleep (Armand et al., 2021). Therefore, we proposed the following hypotheses:

*Hypothesis 2*: Anxiety is negatively related to flourishing.

*Hypothesis 3*: Flourishing is negatively related to sleep disorders.

*Hypothesis 4*: Flourishing will mediates the relationship between anxiety and sleep disorders.

### 2.3 The moderating role of neuroticism

In addition to anxiety, flourishing may also be influenced by neuroticism. The concept of neuroticism was first introduced by the psychologist Eysenck in his theory of personality, as a counterpart to emotional stability (Eysenck and Himmelweit, 1946; Liu and Yang, 2019). People with a high tendency to experience neuroticism are typically described as having a neurotic personality (McCrae and Sutin, 2018). Neuroticism has been found to be the most significant predictor of wellbeing, encompassing factors such as life satisfaction, positive affect and flourishing (Asquith et al., 2022).

According to the Goal Content Theory within the Self-Determination Theory, achieving intrinsic goals can enhance wellbeing, while achieving extrinsic goals has little effect on wellbeing (Ntoumanis et al., 2021). Neuroticism is associated with pessimism, negative self-cognition, and a lower perception of social support (Liu and Yang, 2019). Accordingly, individuals may find it more difficult to achieve intrinsic goals, meaning that they pursue external goals such as increasing their economic income and maintaining an attractive appearance to relieve their internal anxiety (Anglim et al., 2020). Unfortunately, the pursuit of these goals may lower their sense of wellbeing. It appears that individuals with high neuroticism may be more likely to experience lower levels of flourishing due to anxiety. Therefore, we proposed the following:

*Hypothesis 5*: Neuroticism will moderates the process through which flourishing mediates the effect of anxiety on sleep disorders; that is, for students with high neuroticism, this mediation effect is stronger.

**Figure 1:**
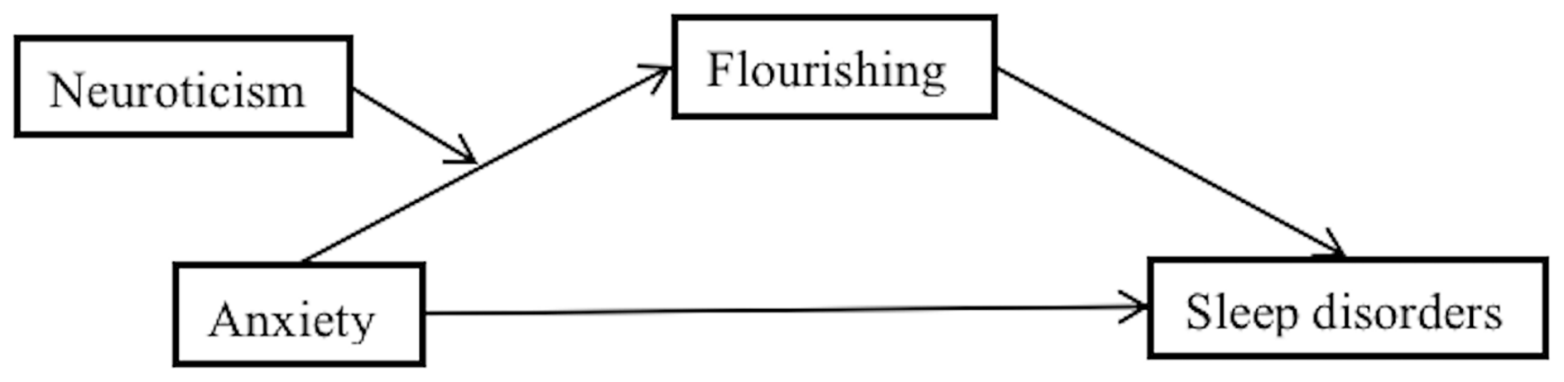
Proposed relationship between anxiety and sleep disorders.

Figure 1 depicts the research model of this study.

## 3 Materials and methods

### 3.1 Participants and procedures

From 2022 to 2023, a cluster sampling method was used to administer a questionnaire survey to students from a university in Guangzhou. A total of 865 medical students were invited to participate in the study, and only 787 submitted questionnaires. However, 27 subjects who chose the same option repeatedly and answered within 250 s were regarded as invalid and deleted. In this survey, eight hundred and sixty-five questionnaires were distributed and 787 questionnaires were collected, of which 760 were deemed valid (effective response rate: 87.86%). The participants consisted of 245 males (32.2% of the overall sample) and 515 females (67.8% of the overall sample), with an average age of 20.54 ± 1.83 years old. Strict confidentiality principles were followed, and all surveys were conducted with the participants’ informed consent. The research design and implementation were compliant with the ethical guidelines outlined in the Helsinki Declaration.

The participants were first given instructions to clarify the purpose of the assessment, the method of answering questions, the voluntary nature of participation, and the principle of anonymity. Then the participants were asked to independently complete the questionnaire within the specified time (1 h), taking into account their own circumstances.

### 3.2 Measures

#### 3.2.1 Anxiety

The Generalized Anxiety Disorder Scale-7 (GAD-7) is a self-assessment scale used to measure the frequency of seven anxiety symptoms experienced in the past 2 weeks (Spitzer et al., 2006). The total score ranges from 0 to 21, with higher scores indicating more pronounced anxiety. Previous studies have demonstrated the reliability and validity of this scale (Spitzer et al., 2006), including in the Chinese population (He et al., 2010). In our study, the GAD-7 showed good reliability (Cronbach’s alpha = 0.94), with a sensitivity of 0.94 and specificity of 0.65 when using a cutoff value of 5 (Plummer et al., 2016).

#### 3.2.2 Sleep disorders

The Pittsburgh Sleep Quality Index (PSQI) is a questionnaire consisting of 18 self-assessment items that measure seven components of sleep disorders: subjective sleep quality, sleep latency, sleep duration, sleep efficiency, sleep disturbances, use of sleep medication, and daytime dysfunction (Liu and Tang, 1996). Each component is scored on a scale from 0 to 3, and the cumulative scores represent the overall sleep quality, with higher scores indicating poorer sleep quality. In our study, the PSQI showed good reliability (Cronbach’s alpha = 0.85).

#### 3.2.3 Flourishing

The Flourishing Scale is an eight-item questionnaire with a 1–7 point scoring method. All items are positively scored, and the score range is 0–56 (Diener et al., 2010). Higher scores indicate greater positive psychological resources and social functioning, reflecting higher levels of flourishing. The revised Chinese version of the Flourishing Scale has demonstrated good internal consistency reliability (Cronbach’s alpha = 0.95). In our study, the Flourishing Scale exhibited good reliability (Cronbach’s alpha = 0.93).

#### 3.2.4 Neuroticism

The Eysenck Personality Questionnaire-Revised Short Scale for Chinese (EPQ-RSC) is a questionnaire consisting of 48 items, including three personality dimension scales and one validity scale (Qian et al., 2000). For this study, we selected 12 items from the neuroticism dimension scale. The neuroticism scale, also known as the emotional stability scale, assesses the degree of emotional instability, with higher scores indicating greater instability. The questionnaire requires participants to respond “yes” or “no” to descriptive statements, with positively scored items receiving 1 point for a “yes” response and 0 points for a “no” response. In our study, the EPQ-RSC neuroticism scale demonstrated good reliability (Cronbach’s alpha = 0.82).

### 3.3 Data analyses

SPSS 27.0 was used to calculate the internal consistencies of the measures, the sample descriptive statistics, correlations among the variables, independent samples t-tests, and regression analysis. Taking Anxiety as the antecedent variable, Flourishing as the intermediary variable, and Sleep disorders as the outcome variable, a hierarchical regression analysis was conducted to test Hypotheses 1, 2 and 3. Taking Anxiety as the grouping variable and Flourishing, and Sleep disorders as the outcome variables, an independent samples t-test was conducted to test the differences in flourishing and sleep disorders among individuals with and without anxiety. To address mediation effect problems, we used the SPSS macro PROCESS 4.1 developed by Hayes (2013) and performed Bootstrap sampling to repeatedly extract 5,000 samples to calculate the 95% confidence interval (CI). We then observed whether the confidence interval of each path included zero and gauged whether the mediation effect was significant. Similarly, the PROCESS macro was used to test the moderating effect of neuroticism. The participants were divided into high/low neuroticism groups to estimate the influence of the mediation effect of flourishing within the different neuroticism groups to test Hypotheses 4 and 5.

## 4 Results

### 4.1 Common method bias test

Given that all of our sample data were collected through self-report, there was a potential for common method bias. To assess this, we carried out Harman’s single-factor test (Tang and Wen, 2020). In our study, the maximum variance explained by the major factor was 30.04%, which was <40% of the critical threshold (Wingate et al., 2018). This indicated that there was no serious common method bias in this study.

### 4.2 Descriptive statistics

The participants consisted of 282 medicine students (37.1% of the overall sample), 317 medical technology students (41.7% of the overall sample), 144 nursing students (18.9% of the overall sample) and 17 others (2.2% of the overall sample). There were differences in anxiety between the different medical specialties (*p* < 0.05), but there were no differences in sleep disorders (*p* = 0.06). The participants included 170 first graders (22.4% of the overall sample), 334 s graders (43.9% of the overall sample), 172 third graders (22.6% of the overall sample) and 84 fourth graders (11.1% of the overall sample). There was no significantly correlation between academic years and anxiety (*p* = 0.09) or sleep disorders (*p* = 0.21). Besides academic years, there were no significantly differences in anxiety (*p* = 0.61) or sleep disorders (*p* = 0.12) between the different genders. Table 1 presents the means and standard deviations of the study variables and correlations between each of the variables, providing preliminary support for the subsequent hypothesis testing.

**Table 1:** Descriptive statistics and correlations among all variables.

	*M*	SD	1	2	3	4
1. Sleep disorders	6.86	3.54	1.00			
2. Flourishing	41.52	8.00	−0.41^**^	1.00		
3. Anxiety	4.33	3.75	0.47^**^	−0.43^**^	1.00	
4. Neuroticism	4.72	3.27	0.42^**^	−0.42^**^	0.56^**^	1.00

*N* = 760, ^*^p < 0.05, ^**^p < 0.01.

### 4.3 Differences in flourishing and sleep disorders among individuals with and without anxiety

Using a cutoff score of 5 on the GAD-7, 336 participants were found to having varying degrees of anxiety (44.21%). Independent samples t-tests showed that there were significant differences in flourishing and sleep disorders ratings between individuals with and without anxiety (*p* < 0.01), as shown in Table 2.

**Table 2:** Differences in flourishing and sleep disorder among individuals with and without anxiety.

Group	Flourishing	Sleep disorders
No anxiety (*n* = 424)	43.89 ± 7.05	5.70 ± 2.93
Anxiety (*n* = 336)	38.53 ± 8.14	8.33 ± 3.69
*t*	−9.55	10.66
*p*	<0.01	<0.01

### 4.4 Mediation effect testing

We further analyzed the 336 participants with varying degrees of anxiety, standardized the variables, and explored the effects of different levels of anxiety on individual sleep disorders and flourishing. The PROCESS version 4.1 macro, model 4, was used to test the mediating effect. The results are shown in Table 3, wherein anxiety significantly and positively predicted sleep disorders (*β* = 0.43, *p* < 0.01), while anxiety significantly and negatively predicted flourishing (*β* = −0.44, *p* < 0.01). When anxiety and flourishing simultaneously predicted sleep disorders, the negative predictive effect of flourishing on sleep disorders was significant (*β* = −0.29, *p* < 0.01), and the positive predictive effect of anxiety on sleep disorders remained significant (*β* = 0.29, *p* < 0.01). The results of the mediation analysis showed that individual flourishing played a mediating role in the relationship between anxiety and sleep disorders, with a mediation effect of 0.13; the 95% Bootstrap confidence interval was [0.07, 0.21], and the mediation effect accounted for 44.83% of the total effect.

**Table 3:** Regression analysis of the mediating role of flourishing.

Regression equation		Overall fit index			Regression coefficient significance			
Outcome variable	Predictor variable	*R*	*R* ^2^	*F*	*β*	LLCI	ULCI	*t*
Sleep disorders	Anxiety	0.31	0.10	36.33^**^	0.43	0.29	0.57	6.03^**^
Flourishing	Anxiety	0.33	0.11	40.81^**^	−0.44	−0.58	−0.31	−6.39^**^
Sleep disorder	Anxiety	0.42	0.17	34.88^**^	0.29	0.16	0.44	4.12^**^
	Flourishing				−0.29	−0.40	−0.19	−5.50^**^

^**^p < 0.01. LLCI, bootstrap 95% lower limit confidence interval; ULCI, bootstrap 95% upper limit confidence interval.

Therefore, Hypotheses 1–4 were supported.

### 4.5 Moderation effect testing

**Figure 2:**
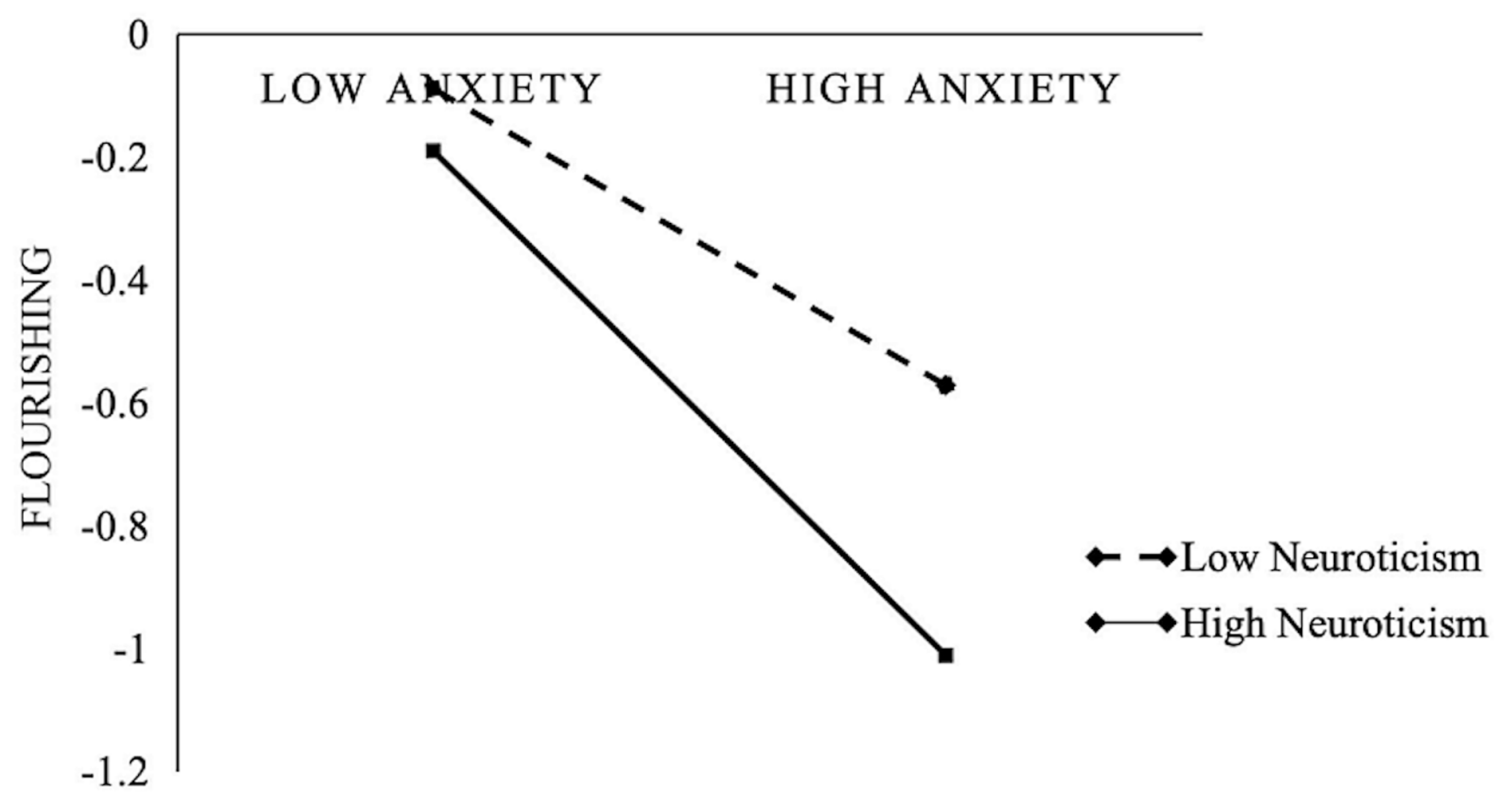
Interaction between anxiety and neuroticism on flourishing.

Given the mediating role of flourishing between anxiety and sleep disorders, we further examined the moderating effect of neuroticism using the PROCESS 4.1 macro, model 7 (Igartua and Hayes, 2021). The results as shown in Table 4, indicated that anxiety significantly and negatively predicted flourishing (*β* = −0.24, *p* < 0.01). Moreover, the interaction term between anxiety and neuroticism significantly and negatively predicted flourishing (*β* = −0.17, *p* < 0.05), suggesting that the impact of anxiety on flourishing is moderated by neuroticism. To further clarify the significance of the neuroticism moderation effect, simple slope analyses were conducted. The results showed that when individuals had higher neuroticism scores (M + SD), the negative predictive effect of anxiety on flourishing was significant (*β* = −0.40, SE = 0.08, *p* < 0.001). On the other hand, when individuals had lower neuroticism scores (M-SD), the negative predictive effect of anxiety on flourishing weakened (*β* = −0.08, SE = 0.13, *p* = 0.552), as illustrated in Table 5 and Figure 2.

**Table 4:** Regression analysis of the moderating effect of neuroticism.

Regression equation		Overall fit index			Regression coefficient significance			
Outcome variable	Predictor variable	*R*	*R^2^*	*F*	*β*	LLCI	ULCI	*t*
Flourishing	Anxiety	0.42	0.17	23.22^**^	−0.24	−0.40	−0.08	−2.98^**^
	Neuroticism				−0.27	−0.38	−0.16	−4.77^**^
	Anxiety × Neuroticism				−0.17	−0.32	−0.02	−2.24^*^

^**^p < 0.01, ^*^p < 0.05. LLCI, bootstrap 95% lower limit confidence interval; ULCI, bootstrap 95% upper limit confidence interval.

**Table 5:** Moderated mediating effect analysis.

Group	Estimated effect	SE	LLCI	ULCI
Lower neuroticism	−0.08	0.13	−0.34	0.18
Higher neuroticism	−0.40	0.08	−0.56	−0.25

LLCI, bootstrap 95% lower limit confidence interval; ULCI, bootstrap 95% upper limit confidence interval.

Therefore, *Hypotheses 5* was supported.

In summary, individual flourishing was found to play a mediating role between anxiety and sleep disorders, and the mediating effect of flourishing was moderated by neuroticism. Specifically, as neuroticism levels increased, the negative predictive effect of anxiety on flourishing intensified, thereby further enhancing the mediating effect on sleep disorders.

## 5 Discussion

Medical students often experience negative emotions such as anxiety due to academic and employment pressures, as well as the challenges of managing interpersonal relationships. They are required to confront unfamiliar knowledge, unfamiliar individuals, and an unpredictable future independently. The pressures of studying, exams, social interactions, and employment can all impact the sleep quality of medical students (Wang and Bíró, 2021). Emotional and sleep issues not only negatively impact daily lives, learning, and social adaptation but also exacerbate the psychological burden placed on medical students. The goals of the current study were to investigate whether flourishing mediated the association between anxiety and sleep disorders and whether neuroticism moderated this association. We constructed a moderated mediation model and tested the mediating role of flourishing and the moderating role of neuroticism in medical college students. The integrated model contributes to our understanding of how anxiety affects sleep quality and under what conditions, offering potential guidance for efforts aiming to help improve emotional wellbeing and quality of sleep in college students.

The results confirmed a positive correlation between anxiety and sleep disorders, with more severe anxiety leading to poorer sleep quality. This points to an important role for anxiety in the development of sleep disorders. Furthermore, anxiety has been found to significantly predict sleep disorders (Ghrouz et al., 2019). Symptoms of anxiety, such as nervousness and autonomic nervous disorders, can cause difficulty falling asleep, shallow sleep, and increased dreaming (Huang and Zhao, 2020). Psychobiological models propose that a state of mental hyperarousal, frequently marked by worry, is a key factor for sleep disorders. Sleep disorders may occur due to a plethora of dysfunctional cognitions, maladaptive behaviors (e.g., excessive time in bed, daytime napping), physiological hyperarousal (e.g., increased nighttime sympathetic activity and decreased parasympathetic activity), and mental hyperarousal (Riemann et al., 2015; Kalmbach et al., 2018). In mental hyperarousal, individuals experiencing sleep disorders often worry about not getting enough sleep and about the consequences of their lack of sleep. As such, their (anxiety-related) beliefs, attitudes and behaviors can contribute to maintenance or worsening of their sleep disorders (Molen et al., 2014).

Moreover, the results of the mediation analysis showed that flourishing plays a mediating role between anxiety and sleep disorders, meaning that anxiety not only directly predicts sleep disorders in college students but can also indirectly predict them through flourishing. Studies on anxiety and flourishing indicate that individuals experiencing uncertain situations and feelings of insecurity may exhibit symptoms of worry, tension, sadness, and anxiety, which may lead to decreased levels of flourishing (Cauberghe et al., 2021; Wasil et al., 2021). Clinical studies on anxiety have also revealed the direct effects of these symptoms physiological factors such as the autonomic nervous system and sleep disorders, leading to uncontrollable tension, restlessness, decreased levels of flourishing, and other negative consequences (Li et al., 2022).

Based on the above, it is apparent that anxiety can lead to a decrease in an individual’s positive psychological resources and lead to a decline in sleep quality by affecting their stable mood and levels of flourishing. Our study suggests that flourishing, as a proximal factor, plays a mediating role in the impact of anxiety on sleep disorders. Sleep disruption is a core feature of anxiety, and anxiety often worsens sleep quality, which suggests a negative cycle involving poor sleep and anxiety. Research studies have indicated dopaminergic hypoactivity in individuals with anxiety who have insufficient sleep (Volkow et al., 2012; Berry et al., 2019). Flourishing could break this negative cycle by increasing dopamine activity (Dfarhud et al., 2014). There is growing interest in enhancing flourishing to mitigate anxiety symptoms and sleep disorders. Future research should explore more positive psychological mediators that bridge the relationship between anxiety and sleep disorders to uncover the specific pathways through which anxiety affects sleep disorders. This study not only examined the partial mediating role of flourishing between anxiety and sleep disorders but also explored the moderating effect of neuroticism on this mediation.

We found that neuroticism moderates the first half of the mediation model. Specifically, when individuals have higher levels of neuroticism, their anxiety has a significant inhibitory effect on flourishing. When individuals have lower neuroticism, the inhibitory effect of anxiety on flourishing is not significant. Compared to individuals with lower neuroticism, the mediating role of flourishing is stronger in individuals with higher neuroticism. In other words, the neurotic personality trait exacerbates the adverse effects of anxiety. Neuroticism plays a moderating role in the impact of anxiety on flourishing, indicating that it acts as a catalyst for reducing flourishing levels in the presence of anxiety. Previous studies on neuroticism have found that individuals with higher neuroticism tend to exhibit more symptoms of worry, leading to lower levels of flourishing. Individuals with high neuroticism may experience strong emotional reactions when facing uncontrollable situations, and if they are unable to handle them through defense mechanisms, they are more likely to develop anxiety and experience lower levels of happiness and flourishing, resulting in more sleep problems (Wasil et al., 2021; Lee et al., 2022). Exercise therapy has been proved to be an effective way to improve neuroticism. By participating in physical exercise and sports activities, individuals can relieve anxiety and tension, enhance self-confidence, and improve emotional stability and sleep quality. Moreover, individuals can adjust their emotions and thinking patterns, increase self-control, and mitigate the impact of neuroticism and sleep disorders through relaxation training, meditation, and mindfulness.

To date, research studies have shown that anxiety and neuroticism interfere with sleep (Chellappa and Aeschbach, 2022). Moreover, most research on sleep disorders and anxiety have focused on negative emotions, while there has been relatively little exploration of the relationship between sleep disorders and flourishing. There is no integrated model to explain the connection between them. Using a moderated mediation model, we explored the impact of anxiety on sleep disorders and further revealed the mediating role of flourishing, as well as the moderating effect of neuroticism. The results not only address how anxiety affects sleep disorders but also shed light on when anxiety may have a stronger or weaker impact on sleep disorders. Specifically, the investigation of the moderating effect of neuroticism confirms that neuroticism exacerbates the mediating effect of anxiety on sleep disorders. Our findings have implications for the prevention and intervention of emotional and sleep difficulties in college students.

### 5.1 Limitations and future research

This study has limitations to be acknowledged. The use of self-report questionnaires to assess sleep disorders and related factors in college students may introduce memory bias or reporting bias. Future research could consider incorporating interviews and hetero-rating scales to comprehensively assess the relationship between the pertinent factors and sleep disorders.

## 6 Conclusion

In conclusion, anxiety not only has a significant and direct predictive effect on sleep disorders but also indirectly predicts sleep disorders through the mediating role of flourishing. The mediating role of flourishing is moderated by neuroticism, and compared to individuals with lower neuroticism, the indirect effect of anxiety on sleep disorder is stronger in individuals with higher neuroticism. The results emphasize the therapeutic necessity of incorporating psychoeducation and strategies that target flourishing and neuroticism in treatments meant to help individuals with anxiety or sleep disorders. In addition, the results suggest that medical schools should pay attention to the positive psychological energy of students and offer regular psychic healing activities.

## Data availability statement

The raw data supporting the conclusions of this article will be made available by the authors, without undue reservation.

## Ethics statement

Ethical review and approval was not required for the study on human participants in accordance with the local legislation and institutional requirements. Written informed consent from the patients/participants or patients/participants’ legal guardian/next of kin was not required to participate in this study in accordance with the national legislation and the institutional requirements.

## Author contributions

CY: Conceptualization, Data curation, Formal analysis, Investigation, Methodology, Software, Visualization, Writing – original draft, Writing – review & editing. ZL: Investigation, Methodology, Software, Writing – review & editing. TS: Data curation, Software, Writing – review & editing, Conceptualization. ZL: Investigation, Software, Writing – review & editing, Data curation. ZJ: Investigation, Writing – review & editing, Methodology, Software. WZ: Investigation, Project administration, Resources, Supervision, Validation, Writing – review & editing, Data curation. ZX: Conceptualization, Funding acquisition, Investigation, Project administration, Resources, Supervision, Validation, Writing – review & editing.
