# Supplementary material for: The effect of anxiety on sleep disorders in medical students: a moderated mediation model
Source: Front Psychol. 2024 Mar 11;15:1338796. doi: 10.3389/fpsyg.2024.1338796 (PMC10961465; doi:10.3389/fpsyg.2024.1338796)
Supplement: Supplementary file 1 [file Data_Sheet_1.docx]

Supplementary Material

The Effect of Anxiety on Sleep Disorders in Medical Students: A Moderated Mediation Model

Chuang Yu, Zhiyi Liu, Tiehong Su, Zhongyu Li, Zinan Jiang, Wen Zhong^*^, Zhongju Xiao^*^

*** Correspondence: Wen Zhong:** [zhong1981@smu.edu.cn,](mailto:xiaozj@smu.edu.cn,) **Zhongju Xiao:** [xiaozj@smu.edu.cn](mailto:xiaozj@smu.edu.cn)

# Supplementary Tables

| **Table 1.** Descriptive statistics and correlations among all variables. | | | | | | |
| --- | --- | --- | --- | --- | --- | --- |
|  | ***M*** | ***SD*** | **1** | **2** | **3** | **4** |
| 1. Sleep disorders | 6.86 | 3.54 | 1.00 |  |  |  |
| 1. Flourishing | 41.52 | 8.00 | −0.41^**^ | 1.00 |  |  |
| 1. Anxiety | 4.33 | 3.75 | 0.47^**^ | −0.43^**^ | 1.00 |  |
| 1. Neuroticism | 4.72 | 3.27 | 0.42^**^ | −0.42^**^ | 0.56^**^ | 1.00 |

Note: *N* = 760, ^*^*P* <0.05, ^**^*P* <0.01.

| **Table 2.** Differences in flourishing and sleep disorder among individuals with and without anxiety. | | |
| --- | --- | --- |
| Group | Flourishing | Sleep disorders |
| No Anxiety（*n* = 424） | 43.89 ± 7.05 | 5.70 ± 2.93 |
| Anxiety（*n* = 336） | 38.53 ± 8.14 | 8.33 ± 3.69 |
| *t* | −9.55 | 10.66 |
| *P* | <0.01 | <0.01 |

| **Table 3.** Regression analysis of the mediating role of flourishing. | | | | | | | | |
| --- | --- | --- | --- | --- | --- | --- | --- | --- |
| Regression equation | | Overall fit index | | | Regression Coefficient significance | | | |
| Outcome variable | Predictor variable | *R* | *R^2^* | *F* | β | LLCI | ULCI | *t* |
| Sleep disorders | Anxiety | 0.31 | 0.10 | 36.33^**^ | 0.43 | 0.29 | 0.57 | 6.03^**^ |
| Flourishing | Anxiety | 0.33 | 0.11 | 40.81^**^ | −0.44 | −0.58 | −0.31 | −6.39^**^ |
| Sleep disorder | Anxiety | 0.42 | 0.17 | 34.88^**^ | 0.29 | 0.16 | 0.44 | 4.12^**^ |
|  | Flourishing |  |  |  | −0.29 | −0.40 | −0.19 | −5.50^**^ |

Note: ^**^*P* <0.01. LLCI:Bootstrap 95% lower limit confidence interval, ULCI:Bootstrap 95% upper limit confidence interval.

| **Table 4.** Regression analysis of the moderating effect of neuroticism. | | | | | | | | |  |  |
| --- | --- | --- | --- | --- | --- | --- | --- | --- | --- | --- |
| Regression equation | | Overall fit index | | | Regression coefficient significance | | | | |  |
| Outcome variable | Predictor variable | *R* | *R^2^* | *F* | β | LLCI | ULCI | *t* |  |  |
| Flourishing | Anxiety | 0.42 | 0.17 | 23.22^**^ | −0.24 | −0.40 | −0.08 | −2.98^**^ |  |  |
|  | Neuroticism |  |  |  | −0.27 | −0.38 | −0.16 | −4.77^**^ |  |  |
|  | Anxiety × Neuroticism |  |  |  | −0.17 | −0.32 | −0.02 | −2.24^*^ |  |  |

Note: ^**^*P* <0.01，^*^*P* <0.05 . LLCI:Bootstrap 95% lower limit confidence interval, ULCI:Bootstrap 95% upper limit confidence interval.

| **Table 5.** Moderated mediating effect analysis. | | | | |
| --- | --- | --- | --- | --- |
| Group | Estimated effect | SE | LLCI | ULCI |
| Lower neuroticism | −0.08 | 0.13 | −0.34 | 0.18 |
| Higher neuroticism | −0.40 | 0.08 | −0.56 | −0.25 |

Note: LLCI:Bootstrap 95% lower limit confidence interval, ULCI:Bootstrap 95% upper limit confidence interval.
